# Use of the Cancer and Aging Research Group Predictive Model for Chemotherapy-Related Toxic Effects in a Multiethnic, Older Adult Asian Population

**DOI:** 10.1001/jamanetworkopen.2022.37196

**Published:** 2022-10-18

**Authors:** Angela Pang, Low Jiali, Alex Ng, Joseph Cheng, Meng Wang, Yean Shin Ng, Yao Yao, Meiling Chun, Francis Ho, Jeremy Tey

**Affiliations:** 1 Department of Haematology Oncology, National University Cancer Institute, Singapore; 2 Yong Loo Lin School of Medicine, National University of Singapore; 3 Department of Medicine, National University Hospital, Singapore; 4 Department of Pharmacy, National University Hospital, Singapore; 5 Department of Surgery, Ng Teng Fong General Hospital, Singapore; 6 Department of Radiation Oncology, National University Cancer Institute, Singapore

## Abstract

**Importance:**

The Cancer and Aging Research Group (CARG) prediction model for chemotherapy-related toxic effects has been developed but not yet validated in older Asian adults. In view of differences in drug metabolism and toxic effect reporting in the Asian population, the ability of this tool to guide the cancer treatment decision-making process in older Asian adults needs to be assessed.

**Objective:**

To examine the validity of the CARG predictive model in a multiethnic Asian cohort of older adults.

**Design, Setting, and Participants:**

In this prognostic study, patients of various Asian ethnicities 70 years or older with a solid tumor diagnosis receiving chemotherapy at the National University Cancer Institute, Singapore, were accrued from June 1, 2017, to January 1, 2019. Their risks of chemotherapy-related toxic effects were calculated using the CARG tool. A geriatric assessment was performed, and the treating oncologist (blinded to the CARG scores) was asked to give an estimated likelihood of toxic effects (low, medium, or high). Chemotherapy-related toxic effects were recorded during each clinic visit. Validation of the prediction model was performed by calculating the area under the receiver operating characteristic curve. Multivariate analyses were performed to identify variables in other domains in the geriatric assessment predicting for severe toxic effects.

**Main Outcomes and Measures:**

Grade 3 to 5 toxic effects and hospitalization.

**Results:**

The study included 200 patients (median age, 74 years [range, 70-89 years]; 110 [55.0%] male; 177 [88.5%] Chinese, 17 [8.5%] Malay, 4 [2.0%] Indian, and 2 [1.0%] other ethnicities [according to Singapore’s national system of race classification]). A total of 137 patients (68.5%) experienced grade 3 to 5 toxic effects, and 131 (65.5%) required hospitalization. The area under the receiver operating characteristic curve for the CARG chemotoxicity prediction model was 0.74 (95% CI, 0.67-0.82), retaining good discrimination in the study population.

**Conclusions and Relevance:**

This prognostic study conducted in a multiethnic Asian cohort of older adults supports the validity of the CARG predictive model in this population, predicting which older adults are at risk of chemotherapy-related toxic effects.

## Key Points

**Question:**

Is the Cancer and Aging Research Group (CARG) chemotherapy toxicity prediction model for older adults applicable in a multiethnic Asian population?

**Findings:**

In this prognostic study, 200 older Asian adults underwent a geriatric assessment, and their risk of chemotherapy-related toxic effects was calculated with the CARG model. The area under the receiver operating characteristic curve for the CARG model was 0.74, indicating that the model retained good discrimination in the study population.

**Meaning:**

The results of this prognostic study support the validity of the CARG prediction model in older Asian adults.

## Teaser

This prognostic study of the Cancer and Aging Research Group prediction model for chemotherapy-related toxic effects assesses the accuracy of this tool for older Asian adults.

## Introduction

Global life expectancy has increased^1,2,3^ along with a disproportionately high incidence of most cancers in older adults. More than 50% of cancer cases occur in people 65 years or older, and these numbers are expected to increase.^4,5^ This demographic trend is also observed in Singapore,^6,7,8^ a multiethnic Asian population.

Providing optimal care for older adults with cancer presents unique challenges.^9^ These challenges include comorbidities, socioeconomic factors,^10^ age-related changes in tumor biology,^11^ and physiological changes with aging that can impact pharmacokinetics and pharmacodynamics of cancer therapies.^12,13^ The effects of these age-related changes in chemotherapeutic dosing and toxic effects have been understudied because most trials have included younger patients and those with good performance status.^14,15^ Identifying areas of vulnerability before treatment initiation is crucial because it allows for implementation of preventive measures, optimizes treatment decisions, and improves outcomes in this population.^16^ A geriatric assessment (GA) is an important tool that measures these independent clinical predictors of morbidity and mortality.

Clinical judgment and performance scales, such as the Karnofsky Performance Status (KPS)^17^ or Eastern Cooperative Oncology Group (ECOG),^18,19^are simple, rapid methods to assess patients’ baseline functional status. These tools may not suffice in identifying patients who are at risk of frailty; hence, a GA before treatment initiation in older adults with cancer is currently recommended by the American Society of Clinical Oncology,^20^ International Society of Geriatric Oncology,^21,22,23,24^ and the National Comprehensive Cancer Network.

A landmark study^25^ in 2011 by the Cancer and Aging Research Group (CARG) established the CARG chemotherapy toxic effects prediction tool based on a prospective cohort to predict grade 3 to 5 chemotherapy-related toxic effects in older adults. This model consists of 11 prechemotherapy variables: age, cancer type, planned chemotherapy dosing, number of chemotherapy drugs, geriatric assessment questions (hearing, falls, instrumental activities of daily living, walking, and social activity), and laboratory values (hemoglobin and creatinine) (Table 1). In this prediction model, risk scores were assigned to each variable. The total risk score ranged from 0 (lowest toxicity risk) to 19 (highest toxicity risk). On the basis of the model, patients were divided into 3 categories based on this risk score: low risk (0-5 points), medium risk (6-9 points), and high risk (10-19 points).

**Table 1.** Cancer and Aging Research Group Prediction Model and Scoring Algorithm for Chemotherapy-Related Toxic Effects^25,26^

Variable	Score
Patient age, y	
≥72	2
<72	0
Cancer type	
GI or GU	2
Other	0
Planned chemotherapy dose	
Standard dose	2
Dose reduced up front	0
Planned No. of chemotherapy drugs	
Polychemotherapy	2
Monochemotherapy	0
Hemoglobin, g/dL	
<11 (Men), <10 (women)	3
≥11 (Men), ≥10 (women)	0
Creatinine clearance, mL/min	
<34	3
≥34	0
How is your hearing? (with a hearing aid, if needed)	
Fair, poor, or totally deaf	2
Excellent or good	0
No. of falls in the past 6 mo	
≥1	3
0	0
Can you take your own medicine?	
With some help/unable	1
Without help	0
Does your health limit you in walking one block?	
Somewhat limited/limited a lot	2
Not limited at all	0
During the past 4 weeks, has your physical health or emotional problems interfered with your social activities (eg, visiting friends or relatives)?	
Limited some of the time, most of the time, or all the time	1
Not limited or minimally limited	0

Abbreviations: GI, gastrointestinal; GU, genitourinary.

SI conversion factor: To convert hemoglobin to grams per liter, multiply by 10.

This model has also been externally validated in the US^26^ and has been shown to have a greater ability to discriminate the risk of chemotherapy toxic effects than KPS. Before our study, the CARG prediction tool had not been tested in a multiethnic Asian population because the original CARG model development and validation cohort consisted of a predominantly White population. In view of the interethnic differences in pharmacokinetics and pharmacodynamics, the CARG model needs to be validated in an Asian population to ensure that the model is applicable. The primary objective of our study was to examine the validity of the CARG tool in older, multiethnic Asian adults. We also assessed the treating oncologists’ estimates of the risks of severe toxic effects.

## Methods

This prognostic study was conducted in Singapore with a multiethnic Asian population. Written informed consent was obtained from each participant. This study was approved by the National University Hospital Institutional Ethics Review Board and followed the Transparent Reporting of a Multivariable Prediction Model for Individual Prognosis or Diagnosis (TRIPOD) reporting guideline.

We aimed to externally validate the CARG chemotherapy toxicity prediction tool in a multiethnic Asian population at the National University Cancer Institute, Singapore (NCIS). Between June 1, 2017, and January 1, 2019, we recruited 200 patients 70 years or older who were diagnosed with a solid tumor and scheduled to receive a new chemotherapy regimen. Each participant’s ethnicity was self-reported, and race categories were defined based on Singapore’s national system of race classification. A designated geriatric oncology nurse administered a GA questionnaire to eligible patients in an outpatient oncology clinic. The GA used in this study covered 7 domains: functional status, cognition, social support, nutrition, psychoemotional, polypharmacy, and comorbidities.

Functional status was assessed using the Katz activities of daily living (ADLs) score (lower scores indicate more need for assistance),^27,28^ the Lawton instrumental ADLs (lower scores indicate more need for assistance),^29^ the KPS (higher scores indicate better physical function),^30^ fall history, presence of visual impairment, presence of hearing impairment, and Timed Up and Go test.^31,32,33,34,35^ Cognitive status was screened using the Mini-Cog assessment tool (scores <3 are suggestive of possible cognitive impairment).^36^ Use of the Mini-Cog tool differs from the original CARG study, which used the Blessed Orientation Memory Score. Social support and activity were assessed using the Medical Outcomes Study–Social Support Survey 4 (MOS-SSS-4)^37^ and Medical Outcome Study–Social Activities Survey 4 (MOS-SAS-4),^38^ respectively. Nutritional status was assessed via changes in weight during 6 months. Psychoemotional status was assessed using the Geriatric Depression Scale 4 (scores of 2 or higher are suggestive of possible depression),^39^ which is different from the Hospital Stress and Anxiety Scale used in the CARG study. Polypharmacy was defined as more than 4 long-term medications and was assessed through a medication review by a trained pharmacist (Y.Y.). Comorbidities were assessed using a patient-reported version of the Older Americans Resources and Services Questionnaire physical health comorbidity subscale.

The information obtained from the GA was inserted into the CARG prediction model along with laboratory values to calculate the patients’ risk of toxic effects. The primary treatment oncologists were concurrently asked to independently estimate the likelihood (low risk, <30%; medium risk, >30%-60%; or high risk, >60%) of chemotherapy-related toxic effects experienced by the patient at the given dose. The oncologists were blinded to the GA results and CARG score.

### Patient Follow-up

Patients were followed up during their entire course of treatment (until 3 months after completion of chemotherapy). Chemotherapy-related toxic effects experienced were recorded at every clinical encounter (standard clinic visits or emergency department visits). Two physicians (the study principal investigator [A.P.] and the treating physician) reviewed the patient’s chemotherapy treatment progress and recorded any grade 3 to 5 chemotherapy-related toxic effects according to the National Cancer Institute Common Terminology Criteria for Adverse Events, version 4.0. Hospitalizations and unexpected visits to the emergency department were also recorded accordingly. Laboratory investigation values were captured as grade 3 to 5 toxic effects on the date of scheduled tests before chemotherapy or at the emergency department visits for chemotherapy-related toxic effects.

### Statistical Analysis

On the basis of the developmental cohort of patients, we know that approximately 55% of older adults develop grade 3 to 4 chemotherapy-related toxic effects. Sample size was calculated based on 80% power to detect a significant difference in the models. We required a minimum of 100 patients who develop grade 3 to 5 toxic effects and 100 who do not. On the basis of the CARG score, patients’ risk of toxic effects was categorized following the cut-off points from the original CARG study as low risk (0-5 points), medium risk (6-9 points), and high risk (10-19 points). Observed grade 3 to 5 toxic effect rates between the groups were compared using a χ^2^ test of proportions. This distribution of toxic effects over the different risk groups was compared with the ability of the primary oncologist’s estimation to predict chemotherapy-related toxic effects using a χ^2^ test of proportions. Logistic regression was performed to identify variables associated with grade 3 to 5 toxic effects. The validity of the model was assessed by composing receiver operating characteristic curves and calculating the area under the curve (AUC). For adverse events outcomes, the proportion of patients experiencing grade 3 or above adverse events was reported. Statistical analysis was performed using Stata software, version 14 (StataCorp LLC). Bonferroni adjustment was performed for multiple comparisons. A 2-sided *P* < .05 was considered to indicate statistical significance.

## Results

The study included 200 patients (median age, 74 years [range, 70-89 years]; 110 [55.0%] male and 90 [45.0%] female; 177 [88.5%] Chinese, 17 [8.5%] Malay, 4 [2.0%] Indian, and 2 [1.0%] other ethnicities [according to Singapore’s national system of race classification]). Median follow-up was 6 months (range, 4-9 months) with no deaths or patients lost to follow-up during the study duration. Table 2 presents the patient demographic characteristics in this study. The most common cancer types were gastrointestinal (80 [40.0%]), lung (38 [19.0%]), and breast (23 [11.5%]) cancers. Eighty-six patients (43.0%) had early-stage cancers, whereas 114 (57.0%) had stage IV cancers. Regarding treatment characteristics, 149 patients (74.5%) received polychemotherapy, 51 (25.5%) received monochemotherapy, 110 (55.0%) received standard-dose chemotherapy, and 90 (45.0%) received reduced-dose chemotherapy.

**Table 2.** Patient and Treatment Characteristics

Characteristic	Patients, No. (%)
**Patient**	
Age group, y	
70-74	109 (54.5)
75-79	63 (31.5)
80-84	22 (11)
≥85	6 (3.0)
Age, mean (SD), y	74.8 (4.26)
Sex	
Female	90 (45.0)
Male	110 (55.0)
Ethnicity	
Chinese	177 (88.5)
Indian	4 (2.0)
Malay	17 (8.5)
Other^a^	2 (1.0)
Cancer type	
Gastrointestinal	80 (40.0)
Lung	38 (19.0)
Breast	23 (11.5)
Gynecologic	14 (7.0)
Genitourinary	12 (6.0)
Other	33 (16.5)
Cancer stage	
I-III	86 (43.0)
IV	114 (57.0)
Educational level	
No education	37 (18.5)
Primary school	86 (43.0)
Secondary school	48 (24.0)
Other	29 (14.5)
Marital status	
Single	9 (4.5)
Married	129 (64.5)
Divorced	12 (6.0)
Widowed	50 (25.0)
Household composition	
Lives alone	12 (6.0)
Lives with spouse, partner, or child	170 (85.0)
Other	18 (9.0)
**Treatment**	
Standard dose	
Yes	110 (55.0)
No	90 (45.0)
No. of chemotherapy drugs	
Monochemotherapy	51 (25.5)
Polychemotherapy	149 (74.5)
Line of chemotherapy	
First line	177 (88.5)
Second line or later	23 (11.5)

^a^ Reported as other according to Singapore’s national system of race classification.

### Geriatric Assessment

The GA variables and results are presented in Table 3. The mean (SD) physician-rated KPS score was 86.25 (13.50), with scores ranging from 50 to 100. The mean (SD) Katz ADLs score was 5.65 (1.04) (range, 0-6), and the mean (SD) Lawton score for instrumental ADLs was 6.13 (1.93) (range, 0-8).

**Table 3.** Geriatric Assessment and Laboratory Values

Assessment	Patients, No. (%)^a^
Functional status, mean (SD)	
Katz ADLs score, mean (SD)^b^	5.65 (1.04)
Lawton IADLs score, mean (SD)^c^	6.13 (1.93)
Karnofsky Performance Scale score, mean (SD)^d^	86.25 (13.5)
History of falls	
0	175 (87.5)
≥1 In the past 6 mo	25 (12.5)
Timed Up and Go result, s	
≤12	31 (15.5)
>12	169 (84.5)
Sensory assessment	
Visual impairment	31 (15.5)
Hearing impairment	25 (12.5)
Unintentional weight loss of ≥10%	127 (63.5)
No. of comorbid conditions	
0	8 (4)
1	5 (2.5)
2	19 (9.5)
≥3	168 (84)
Cognition (Mini-Cog test score)^e^	
≥3	175 (87.5)
<3	25 (12.5)
Emotional well-being (Geriatric Depression Scale 4 score)^f^	
<2	186 (93.0)
≥2	14 (7.0)
Social support	
Social activity affected by physical or mental health (Medical Outcome Study–Social Activity Survey score)	91 (45.5)
Inadequate social support (4-Item Medical Outcome Study–Social Support Survey)	30 (15.0)
Hemoglobin	
<10 g/dL (Women), <11 g/dL (men)	55 (27.5)
≥10 g/dL (Women), ≥11 g/dL (men)	145 (72.5)
Creatinine clearance, mL/min	
<34	12 (6)
≥34	188 (94)

Abbreviations: ADL, activities of daily living; IADL, instrumental activities of daily living.

SI conversion factor: To convert hemoglobin to grams per liter, multiply by 10.

^a^ Data are presented as number (percentage) of patients unless otherwise indicated.

^b^ For Katz scores, lower scores indicate more need for assistance.

^c^ For Lawton scores, lower scores indicate more need for assistance.

^d^ For Karnofsky Performance Scale scores, higher scores indicate better physical function.

^e^ For the Mini-Cog test, scores less than 3 are suggestive of possible cognitive impairment.

^f^ For the Geriatric Depression Scale 4, scores of 2 or higher are suggestive of possible depression.

Twenty-five patients (12.5%) had falls during the preceding 6 months, and 50 patients (25%) required an assistive device for ambulation. A total of 169 patients (84.5%) required more than 12 seconds to perform the Timed Up and Go test.^34,35^

One hundred and twenty-seven patients (63.5%) had experienced unintentional weight loss of more than 10% during the past 6 months. Ninety patients (45.5%) were taking 4 or more long-term medications before the commencement of chemotherapy. A total of 168 (84.0%) had 3 or more preexisting comorbidities. Fourteen patients (7.0%) scored 2 or more on the Geriatric Depression 4 questionnaire, and 25 (12.5%) scored less than 3 on the Mini-Cog screening test, suggesting a high likelihood of cognitive impairment. Ninety-one patients (45.5%) felt that there was an impact on their social activity because of their health in the MOS-SAS-4, and 30 (15.0%) felt that they did not have adequate social support (defined by none to some of the time) in the affectionate domain or had a lack of social interaction, emotional, or tangible support on the MOS-SSS-4.

### Chemotherapy-Related Toxic Effects

Of the 200 patients, 137 (68.5%) experienced at least 1 grade 3 to 5 toxic effect, and 131 (65.5%) were hospitalized for chemotherapy-related toxic effects (Table 4). The most common grade 3 to 4 toxic effects observed were neutropenia (74 [37.0%]), anemia (55 [27.5%]), hyponatremia (46 [23.0%]), pneumonia (44 [22.0%]), and hypokalemia (25 [12.5%]). No treatment-related mortality was observed.

**Table 4.** Chemotherapy-Related Toxic Effects Observed in the 200 Study Patients

Toxic effect type	Total No. (%)	No. (%) by toxic effect grade		
		Grade 3	Grade 4	Grade 5
Any grade 3-5 toxic effects	137 (68.5)	131 (65.5%)	32 (16%)	0
Hematologic toxic effects				
Neutropenia	74 (37.0)	44 (22.0)	30 (15)	0
Anemia	55 (27.5)	55 (27.5)	0	0
Thrombocytopenia	6 (3.0)	3 (1.5)	3 (1.5)	0
Nonhematologic toxic effects				
Hyponatremia	46 (23.0)	40 (20.0)	6 (3.0)	0
Pneumonia	44 (22.0)	35 (17.5)	9 (4.5)	0
Hypokalemia	25 (12.5)	20 (10.0)	5 (2.5)	0
Colitis or genitourinary	14 (7.0)	12 (6.0)	2 (1.0)	0
Dehydration	14 (7.0)	13 (6.5)	1 (0.5)	0
Urinary tract infection	14 (7.0)	14 (7.0)	0	0
Hypophosphatemia	13 (6.5)	13 (6.5)	0	0
Mucositis	11 (5.5)	10 (5.0)	1 (0.5)	0
Diarrhea	11 (5.5)	11 (5.5)	0	0
Nausea or vomiting	10 (5.0)	10 (5.0)	0	0
Loss of appetite	9 (4.5)	8 (4.0)	1 (0.5)	0

### CARG Model Validation

The CARG model risk score ranges from 0 to 19 points and is divided into 3 groups (low risk, 0-5 points; medium risk, 6-9 points; and high risk, 10-19 points). Forty-five patients (22.5%) were classified as low risk, 101 (50.5%) as medium risk, and 54 (27.0%) as high risk. In our study cohort, a statistically significant increase in toxic effect risk was found with an increasing risk score (28 [62.2%] in the low-risk group, 64 [63.4%] in the medium-risk group, and 45 [83.3%] in the high-risk group; *P* = .02). We found that a 1-point increase in total score was associated with a 15% increase in the odds of developing grade 3 to 5 toxic effects (odds ratio [OR], 1.15; 95% CI, 1.03-1.28; *P* = .01).

Multivariate analysis showed that patients with gastrointestinal and genitourinary cancers had a lower odds of developing grade 3 to 5 toxic effects (OR, 0.59; 95% CI, 0.42-0.83; *P* = .03), whereas patients with low hemoglobin levels (<10 g/dL for women and <11 g/dL for men [to convert to grams per liter, multiply by 10]) had a higher odds of developing grade 3 to 5 toxic effects (OR, 1.60; 95% CI, 1.21-2.14; *P* = .01) (Table 5). The AUC for the CARG predictive model in our study (eFigure in the Supplement) was 0.74 (95% CI, 0.67-0.82), indicating that the model retained good discrimination.

**Table 5.** Univariate and Multivariate Analyses of the Cancer and Aging Research Group Chemotherapy Toxicity Prediction Model

Characteristic	Grade 3-5 toxic effects		Univariate		Multivariate		
	Yes	No	OR (95% CI)	*P* value	OR (95% CI)	*P* value
Age >72 y	104	43	1.21 (0.87-1.68)	.26	1.31 (0.90-1.91)	>.99
Gastrointestinal or genitourinary cancer (referent, other cancers)	58	39	0.67 (0.50-0.91)	.01	0.59 (0.42-0.83)	.03
Chemotherapy dosing (referent, reduced dose)	78	32	1.13 (0.84-1.53)	.42	1.35 (0.95-1.91)	.99
No. of chemotherapy drugs (referent, monochemotherapy)	104	45	1.12 (0.80-1.57)	.50	1.07 (0.74-1.55)	>.99
Hemoglobin (<10 g/dL in women, <11 g/dL in men)	46	9	1.44 (1.11-1.88)	.006	1.60 (1.21-2.14)	.01
Creatinine clearance (<34 mL/min)	10	2	1.34 (0.80-2.24)	.27	1.60 (0.89-2.85)	>.99
Hearing, fair or worsened	16	7	1.03 (0.64-1.65)	.91	0.82 (0.48-1.40)	>.99
No. of falls in the last 6 mo (referent, ≥1 fall)	18	6	1.13 (0.81-1.56)	.47	1.10 (0.74-1.52)	>.99
IADL, taking medicine with some help or unable	16	6	1.26 (0.47-3.38)	.65	0.60 (0.19-1.94)	>.99
Medical Outcomes Study						
Walking 1 block somewhat limited/a lot	43	12	1.39 (0.97-2.00)	.07	1.23 (0.81-1.87)	>.99
Decreased social activity due to physical or emotional health	83	31	1.59 (0.87-2.89)	.13	1.54 (0.77-3.10)	>.99

Abbreviations: IADL, instrumental activities of daily living; OR, odds ratio.

SI conversion factor: To convert hemoglobin to grams per liter, multiply by 10.

### Oncologists’ Prediction of Toxic Effects

The patients’ primary oncologists predicted that 55 patients (27.5%) were at low risk, 117 (58.5%) were at medium risk, and 28 (14.0%) were at high risk of developing grade 3 to 5 toxic effects. The incidence of grade 3 to 5 toxic effects was 51.0% in the predicted low risk group, 74.0% in the medium risk group, and 82.0% in the high-risk group. The AUC for the oncologists’ prediction was 0.63 (95% CI, 0.55-0.70), which has a lower discriminatory value compared with the AUC of the CARG prediction model at 0.74.

## Discussion

Treatment decisions in oncology are mostly based on clinical judgment and performance scales, such as the KPS. However, additional factors that can be identified in a GA, such as comorbidities, functional ability, nutritional status, cognition, psychoemotional health, and social support, must be considered to direct individual treatment strategies to optimize outcomes in older adults with cancer.^40^ Geriatric assessment–directed interventions have been shown to reduce treatment-related toxic effects^41,42,43^ and improve quality of life in studies performed in tertiary cancer centers with geriatric oncology services, as well as in community oncology practices with tailored GA and management recommendations.

The CARG toxicity prediction tool developed by Hurria et al^25^ was derived from various GA domains, coupled with treatment characteristics and laboratory values. This chemotoxicity prediction tool serves as an aid to oncologists who treat older adults with cancers and leads to the reduction of treatment-related toxic effects.

Other studies have evaluated the utility of the CARG model outside the US.^44,45,46^ Within the continent of Asia, Ostwal et al^44^ have validated the utility of the CARG model in the Indian population. Suto et al^45^ have similarly found that the CARG model predicted chemotherapy-related toxic effects in a small (<100 patients) retrospective study conducted in a Japanese population, whereas a prospective validation study^46^ of the CARG performed in Hong Kong had negative results, which may be attributed to the inclusion of patients who received only targeted therapy and no chemotherapy in the study population.

To our knowledge, our study is the first prospective study to validate the utility of the CARG toxicity prediction tool in a multiethnic Asian population. The AUC for the CARG model in this study was 0.74, which is similar to the original study (AUC = 0.72) and higher than the validation study by the CARG group (AUC = 0.65).

The treating oncologists’ estimates of chemotherapy-related toxic effect risk did predict severe chemotherapy-related toxic effects (AUC = 0.63) but with a lower discriminatory value than the CARG prediction model (AUC = 0.74). To our knowledge, 2 other studies^47,48^ have evaluated the predictive value of treating oncologists’ estimates of severe toxic effects, and both had negative results. However, this prediction is subjective, and the estimation of participating oncologists may be biased by the knowledge that the patients were enrolled in this chemotoxicity study.

In our study, we observed that the risk of chemotherapy toxicity was high in older adults with cancer in the Asian population. More than half of our patients experienced grade 3 to 5 toxic effects and required hospitalization. These results are comparable to those of the CARG toxicity tool development and validation cohort.^26,44^

Of interest, the risk of chemotherapy-related toxic effects in patients with gastrointestinal or genitourinary cancers was reduced in our study. This finding differs from the original CARG developmental cohort, in which gastrointestinal or genitourinary cancers predicted an increased risk of chemotherapy-related toxic effects. This finding may be attributable to a higher proportion of our gastrointestinal or genitourinary cancer population being patients with colorectal and gastric cancers (n = 71) who had received fluoropyrimidine-based chemotherapy. Previous retrospective analyses have suggested that differences exist in the tolerability of fluoropyrimidines, with many reports indicating that East Asian patients experience a lower incidence of serious toxic effects when treated with fluorouracil-based or capecitabine-based regimens compared with White patients.^49,50,51^ This finding may explain the lower incidences of chemotherapy-related toxic effects in the patients with gastrointestinal or genitourinary cancers in our study.

Before our study, the validity of the CARG toxicity prediction tool in Asians was uncertain because Asians made up only 5% of the population in the CARG toxicity tool development and validation cohort.^25,26^ With pharmacoethnicity^52,53^ recognized as an important factor accounting for interracial variation in anti–cancer drug toxicity owing to allelic variants of genes encoding expressed drug-metabolizing enzymes, we would expect there to be differences in drug metabolism, with resultant differences in incidences of toxic effects among different ethnic groups.^54,55^ Despite the differences in the study population, we have validated the use of the CARG prediction tool in a multiethnic Asian population and found that it can be effectively used to identify older Asian patients who are at an increased risk of developing grade 3 to 5 toxic effects.

### Future Directions

Future directions would include expanding to multiple centers in Asia and evaluating the model for specific tumor types. Longitudinal evaluation of toxic effects in older adults will also guide management and aid in the reduction of chemotherapy-related morbidities in Asian patients of different ethnicities. The development of a model that predicts toxic effects in patients who require biologics or immunotherapy with or without chemotherapy in various tumor types is essential. The development of artificial intelligence to predict toxic effects of various treatment permutations may also complement treatment decision-making.

### Limitations

Our study has certain limitations. This is a single-center study, so the results may not be generalizable across the rest of Asia. In addition, our study population consisted predominantly of patients of Chinese ethnicity; hence, other ethnic groups may be underrepresented, although the sample is consistent with Singapore’s population ethnicity makeup.^56^

Although the CARG model can identify patients at high risk of chemotherapy-related toxic effects with a 1-point increase translating to a 15% increase in the risks of grade 3 to 5 toxic effects, it was less able to discriminate between moderate and high risks. This model also does not consider multiple grade 1 to 2 toxic effects, which may similarly result in the modification or discontinuation of treatment.^57^ It is important to consider that premature treatment termination has been reported to affect cancer-related survival.^58^

Next, as in the development and validation of the CARG studies, hematologic malignant neoplasms were excluded in our study, and the model is currently applicable only in solid tumors. This model also does not predict toxic effects from biologics^59,60,61^ or immunotherapy^62,63,64^ even though these agents have dramatically altered the landscape of cancer treatment. Another limitation of the CARG model is that it is used primarily to evaluate an older patient’s strengths and vulnerabilities before the start of a new chemotherapy regimen, which provides a snapshot that is likely to change along the disease trajectory.

## Conclusions

This prognostic study conducted in a multiethnic Asian cohort in Singapore provides supporting evidence that the CARG predictive model is equally applicable in an Asian population, predicting which older adults are at risk of chemotherapy-related toxic effects. The CARG model may serve as a useful tool to guide the treatment decision-making process for patients and their oncologists.

## Notes

Supplement. **eFigure.** Receiver Operating Characteristic Curve (ROC) for the CARG Predictive Model
