# Supplementary material for: Use of the Cancer and Aging Research Group Predictive Model for Chemotherapy-Related Toxic Effects in a Multiethnic, Older Adult Asian Population
Source: JAMA Netw Open. 2022 Oct 18;5(10):e2237196. doi: 10.1001/jamanetworkopen.2022.37196 (PMC9579905; doi:10.1001/jamanetworkopen.2022.37196)
Supplement: Supplement. — eFigure. Receiver Operating Characteristic Curve (ROC) for the CARG Predictive Model [file jamanetwopen-e2237196-s001.pdf]

Supplementary Online Content

Pang A, Jiali L, Ng A, et al. Use of the Cancer and Aging Research Group predictive model for chemotherapy-related toxic effects in a multiethnic, older adult Asian population. *JAMA Netw Open*. 2022;5(10):e2237196. doi:10.1001/jamanetworkopen.2022.37196

eFigure. Receiver Operating Characteristic Curve (ROC) for the CARG Predictive Model

This supplementary material has been provided by the authors to give readers additional information about their work.

© 2022 Pang A et al. *JAMA Network Open.*

eFigure. Receiver Operating Characteristic Curve (ROC) for the CARG Predictive Model

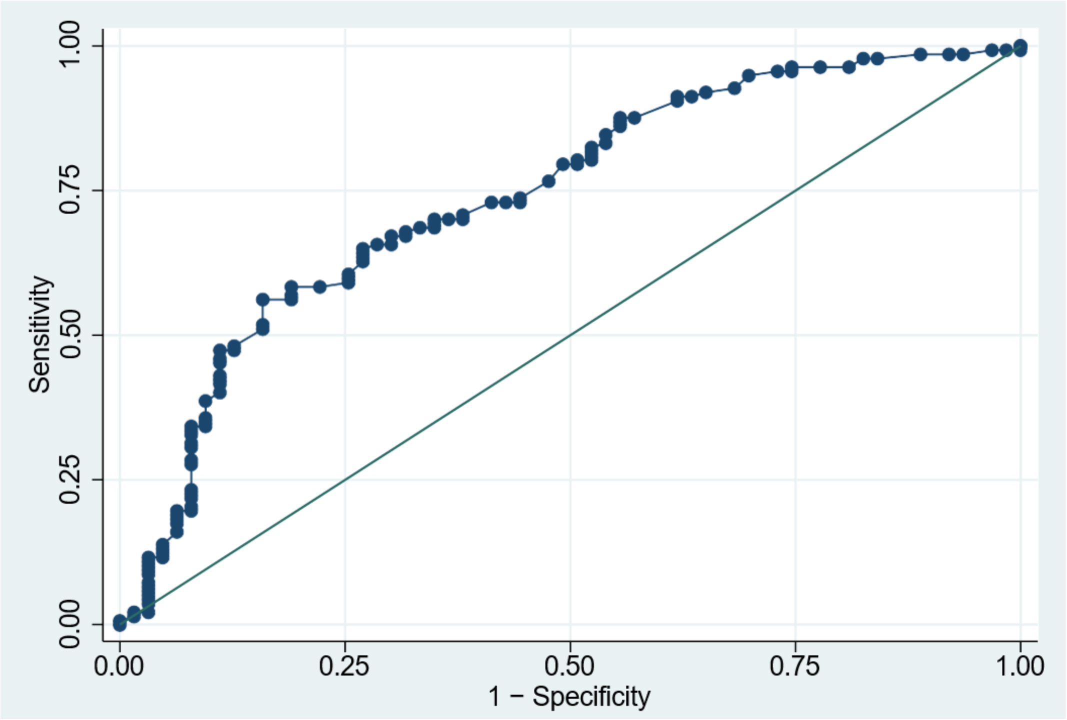

Area under the ROC curve = 0.7411

© 2022 Pang A et al. *JAMA Network Open.*
